# Social network position is a major predictor of ant behavior, microbiota composition, and brain gene expression

**DOI:** 10.1371/journal.pbio.3002203

**Published:** 2023-07-24

**Authors:** Tomas Kay, Joanito Liberti, Thomas O. Richardson, Sean K. McKenzie, Chelsea A. Weitekamp, Christine La Mendola, Matthias Rüegg, Lucie Kesner, Natasha Szombathy, Sean McGregor, Jonathan Romiguier, Philipp Engel, Laurent Keller

**Affiliations:** 1 Department of Ecology and Evolution, University of Lausanne, Lausanne, Switzerland; 2 Department of Fundamental Microbiology, University of Lausanne, Lausanne, Switzerland; 3 School of Biological Sciences, University of Bristol, Bristol, United Kingdom; 4 Department of Biology and Ecology, University of Montpellier, Montpellier, France; Queen Mary University of London, UNITED KINGDOM

## Abstract

The physiology and behavior of social organisms correlate with their social environments. However, because social environments are typically confounded by age and physical environments (i.e., spatial location and associated abiotic factors), these correlations are usually difficult to interpret. For example, associations between an individual’s social environment and its gene expression patterns may result from both factors being driven by age or behavior. Simultaneous measurement of pertinent variables and quantification of the correlations between these variables can indicate whether relationships are direct (and possibly causal) or indirect. Here, we combine demographic and automated behavioral tracking with a multiomic approach to dissect the correlation structure among the social and physical environment, age, behavior, brain gene expression, and microbiota composition in the carpenter ant *Camponotus fellah*. Variations in physiology and behavior were most strongly correlated with the social environment. Moreover, seemingly strong correlations between brain gene expression and microbiota composition, physical environment, age, and behavior became weak when controlling for the social environment. Consistent with this, a machine learning analysis revealed that from brain gene expression data, an individual’s social environment can be more accurately predicted than any other behavioral metric. These results indicate that social environment is a key regulator of behavior and physiology.

## Abstract 2

This study combines automated behavioral tracking of ants with multi-omics, revealing that individual behavior, brain gene expression, and gut microbiota composition are all more strongly correlated with social network position than they are with one another, indicating that social interactions likely regulate social behavior and underlying physiology.

## Introduction

In highly social species, physiology and behavior are profoundly and reciprocally intertwined with social environments. Studies in a variety of species have shown intricate links between the social environment and gene expression [1–3], microbiota composition [4], and behavior [5,6]. However, as is typical in complex biological systems, redundant correlations are ubiquitous: Microbiota composition correlates with behavior [4,7–10] and gene expression [11,12], and gene expression is linked to behavior [13,14] and a plethora of other traits. Further, the social environment is often confounded by the physical environment and demographic processes [15–17]. Teasing apart the correlation structure among these variables has therefore been challenging. Moreover, most studies have focused on one or few variables, and the resolution of the social environmental data has been limited.

Social insect colonies are highly tractable systems for studying the relationships between organismal biology and the social environment [18]. They typically show marked division of labor with individuals within the colony specializing in behaviors such as nursing the brood or foraging [19]. Individuals interact most frequently with other individuals performing the same behavior, leading to behavior-associated community structure in the colony social network [16,20]. Young individuals typically nurse, and with age, they transition to foraging [21–27]. Both behavior and age are associated with brain gene expression [28,29] and microbiota composition [30,31]. Here, we combine automated behavioral tracking with a multiomic approach to simultaneously investigate the correlation structure among social environment, physical environment, behavior, age, brain gene expression, and gut microbiota composition. We used the carpenter ant *Camponotus fellah* as a model system because the social environment of this species is well characterized and the associations between social environment, age, and foraging behavior have already been quantified [16,20].

## Results and discussion

**Fig 1:**
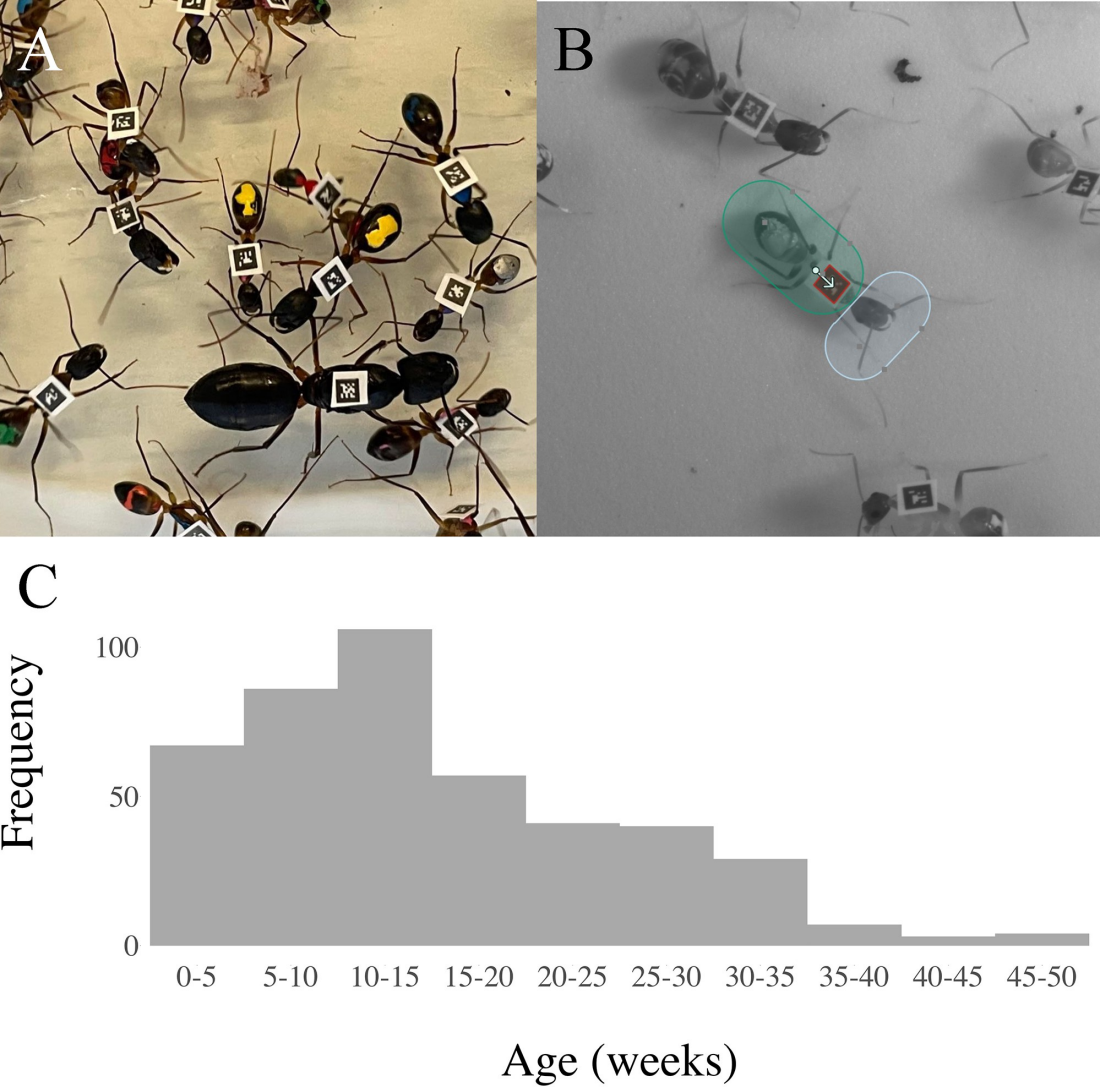
System overview. (**A**) Ants were tagged with unique 1.4 mm^2^ matrix barcodes and paint-marked to indicate their age. (**B**) Head and body regions were defined around each tag. (**C**) Worker age distribution across the 4 colonies. See Fig A in S1 Text for equivalent distributions per colony. The code and data used in this figure are available on Zenodo (doi.org/10.5281/zenodo.8043085 - data: “Fig 1C&S1.csv”; code: “02-Main.R”).

We tracked 4 queenright colonies each containing approximately 100 known-age workers (Fig 1). From the automated tracking data, we inferred all pairwise social interactions, determined the spatial distributions of all individuals, and quantified 6 of the most frequent and identifiable task behaviors (tending the queen, foraging, nursing, guarding, trophallaxing, and cleaning). Immediately after behavioral tracking, RNA-sequencing was performed on whole individual brains, and 16S rRNA gene sequencing was performed on surface-sterilized individual abdomens.

**Fig 2:**
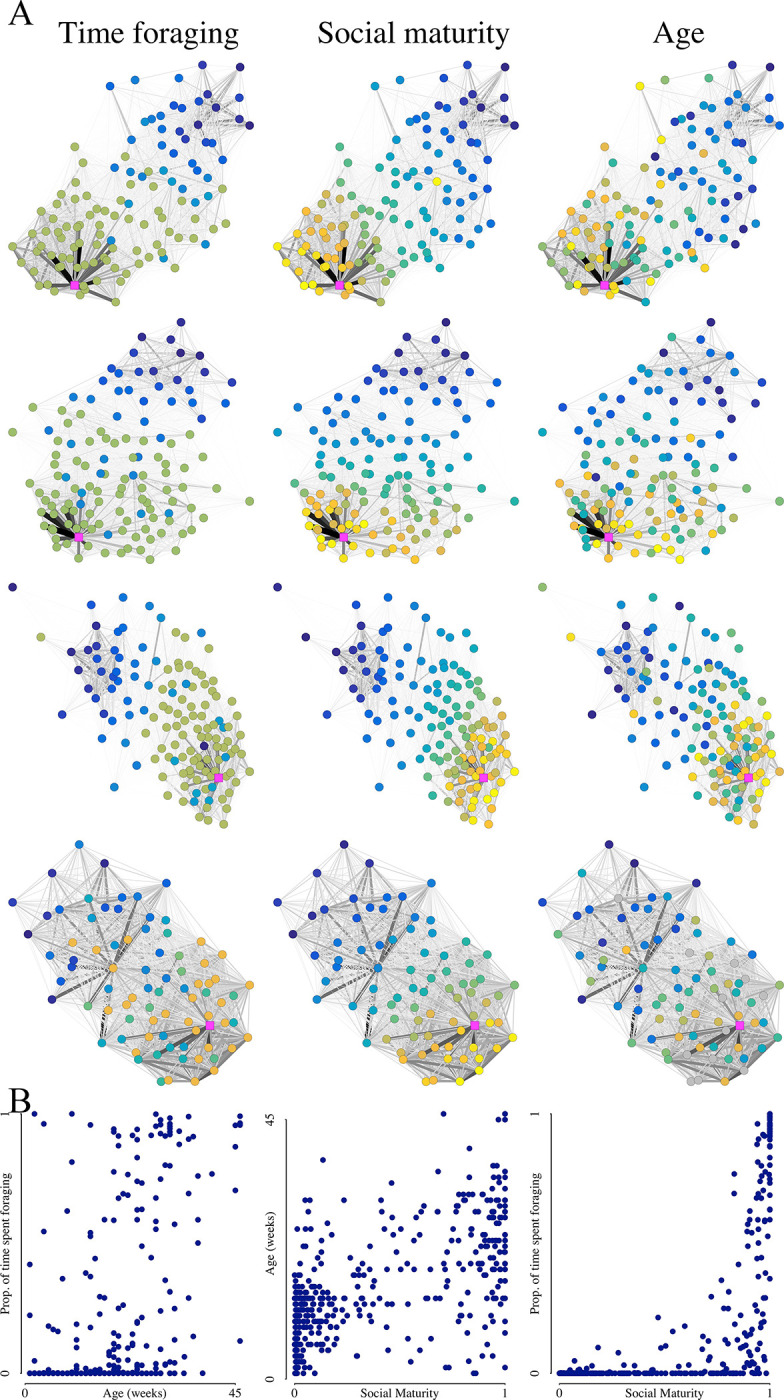
Social network position, time spent foraging, and age. (**A**) The social networks for each of the 4 colonies (rows) with workers colored according to time spent foraging (column 1), social maturity (column 2), and age (column 3). Lowest values are yellow; highest values are dark blue. Queens are colored magenta. Edge color intensity and width correspond to edge strength. Layouts are calculated with the Fruchterman–Reingold algorithm [32] using R package “iGraph” [33]. (**B**) Scatter plots relating proportion of time spent foraging, social maturity, and age. The code and data used in this figure are available on Zenodo (doi.org/10.5281/zenodo.8043085 - data: all 4 “Fig 2A…” csv files and “Fig 2B.csv”; code: “02-Main.R”).

*C*. *fellah* social networks comprise 2 overlapping communities (groups within which individuals interact frequently and between which individuals interact rarely); one comprising individuals that tend to interact with the queen and brood and the other comprising individuals that tend to leave the nest to forage [20]. Individual position in the social network can be described with “social maturity,” a community detection–based metric that ranges from 0 to 1 and that quantifies the extent to which individuals are associated with the nurse versus the forager social community (see Materials and methods and [20]). Consistent with previous results, social maturity was positively correlated with age and the proportion of time spent foraging (Fig 2; social maturity versus age linear mixed effects regression (LMER) with colony identity as a random factor: R^2^ = 0.483, t = 20.23, *p <* 0.001. Social maturity versus proportion of time spent foraging LMER with colony identity as a random factor: R^2^ = 0.528, t = 22.45, *p <* 0.001).

**Fig 3:**
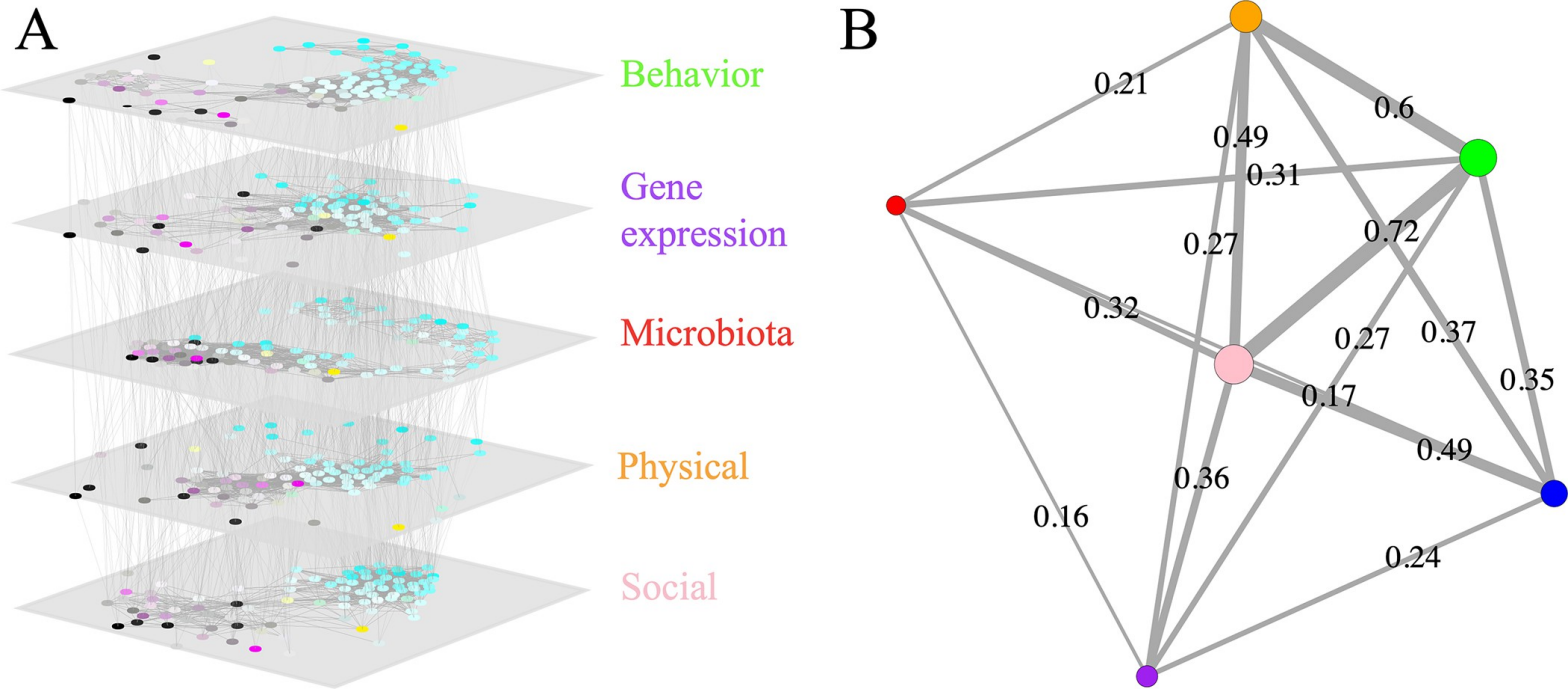
Similarity in social network position, physical environment, microbiota, brain gene expression, behavior, and age. (**A**) A 5-layer multiplex network constructed from behavior, brain gene expression, microbiota, the physical environment, and social interactions. In each layer, each node represents a worker. Nodes are colored according to behavior (cyan = nursing; yellow = cleaning; magenta = foraging; black = guarding). Intralayer edges are unweighted and connect pairs whose interaction strength exceeds the upper quartile of the edge–weight distribution. Interlayer edges connect each worker with itself in the adjacent layers. (**B**) Graphical representation of the correlation (R^2^ values) between the 5 layers and age (in blue). Edge width is proportional to edge strength. Layout is calculated with the Fruchterman–Reingold algorithm [32], and vertices are colored according to the layer labels in panel (**A**) and sized according to their strength (i.e., the sum of their weighted connections). The code and data used in this figure are available on Zenodo (doi.org/10.5281/zenodo.8043085 - data: all 11 “Fig 3A…” txt files and “Fig 3B.csv”; code: “Multiplex.py” and “04-InterlayerCorr.R”).

To examine the relationships between brain gene expression profile, microbiota composition, physical environment, social network position, and behavioral profile, we first constructed 5-layer multiplex networks (Fig 3A). In this approach, nodes represent workers and intralayer edges represent pairwise interaction frequency in the social layer, or pairwise similarity (measured by Euclidean distance between profiles) in other layers. Multiple layers show different types of relationships between the same nodes. Inspection of these multiplex networks revealed striking similarities between layers. Individuals with similar behavior also exhibited similar brain gene expression profiles, microbiota compositions, occupied similar physical environments, and social maturities. To compare the strength of the relationships between these 5 layers and age, we reduced each layer to a single dimension (using social maturity for the social layer, and principal component analysis (PCA) for the other layers). We calculated R^2^ values between these 6 variables and represent the correlations in network form (Fig 3B; values are averages across the 4 colonies; See Fig B in S1 Text for equivalent plots per colony). In this “network-of-networks,” social maturity stands out as a central “hub.” All of the other variables were more correlated with social maturity than with any other variable, except physical environment, which was best correlated with behavior and second best with social maturity. Importantly, both physiological measures (gut microbiota and brain gene expression) were considerably more correlated with social maturity than with behavior, age, or physical environment. The average R^2^ value between brain gene expression and social maturity was 0.36, 33% greater than the average R^2^ values between brain gene expression and the physical environment or behavior, and 50% greater than the average R^2^ value between brain gene expression and age. Similarly, the average R^2^ value between microbiota composition and social maturity was 0.32, 3% greater than the average R^2^ value between microbiota composition and behavior, 52% greater than the average R^2^ value between microbiota composition and physical environment, and 88% greater than the average R^2^ value between microbiota composition and age.

The strength of the relationship between brain gene expression and social maturity is of particular interest because it implies that social interactions may have a direct and considerable effect on brain function (i.e., that their association is not an indirect consequence of brain gene expression being associated with behavior or age). Because the PCA of gene expression data could be strongly influenced by few highly expressed genes, we next used differential gene expression analyses to investigate the number of genes differentially expressed by behavior, physical environment, age, microbiota composition, and social maturity. Consistent with the previous analysis, social maturity was associated with the differential expression of the highest number of genes (33% of genes, on average across colonies). Individual behavioral profile was associated with the differential expression of 30% of genes, physical environment with 29% of genes, age with 27% of genes, and microbiota composition with 13% of genes (Table 1). This global pattern was independently true within each colony, meaning that the number of genes differentially expressed as a function of social maturity was significantly higher than the number of genes differentially expressed by behavior in a paired *t* test (*p* = 0.024; see Fig C in S1 Text for the percentage of genes differentially expressed by each variable in each colony). This difference became even greater when considering the number of genes differentially expressed by each variable when controlling for each other variable. When controlling for behavior, social maturity still explained the differential expression of 7.3% of genes, whereas when controlling for social maturity, behavior explained the differential expression of only 0.034% of genes. This pattern was also independently true for all 4 colonies (with *>*10-fold differences in all colonies; see Fig C in S1 Text for full colony-level analyses), strongly supporting the notion that brain transcriptomic variation is more linked to social network position than to behavior.

**Table 1:** The number of genes differentially expressed (out of a total of 14,664 genes) in the brain by each of social maturity, behavior, age, the physical environment and gut microbiota composition (column 1), and the percentage of that number that remain differentially expressed when controlling for each other variable (columns 2–6). Few genes remain differentially expressed by behavior, age, the physical environment, or microbiota composition when controlling for the social maturity.

Variable	Without control	Social (%)	Behavior (%)	Age (%)	Physical (%)	Microbiota (%)
Social	3,581	-	18	46	37	79
Behavior	3,125	1	-	39	28	67
Age	2,810	8	19	-	30	70
Physical	3,007	0.5	6	35	-	75
Microbiota	1,128	2	2	11	13	-

**Fig 4:**
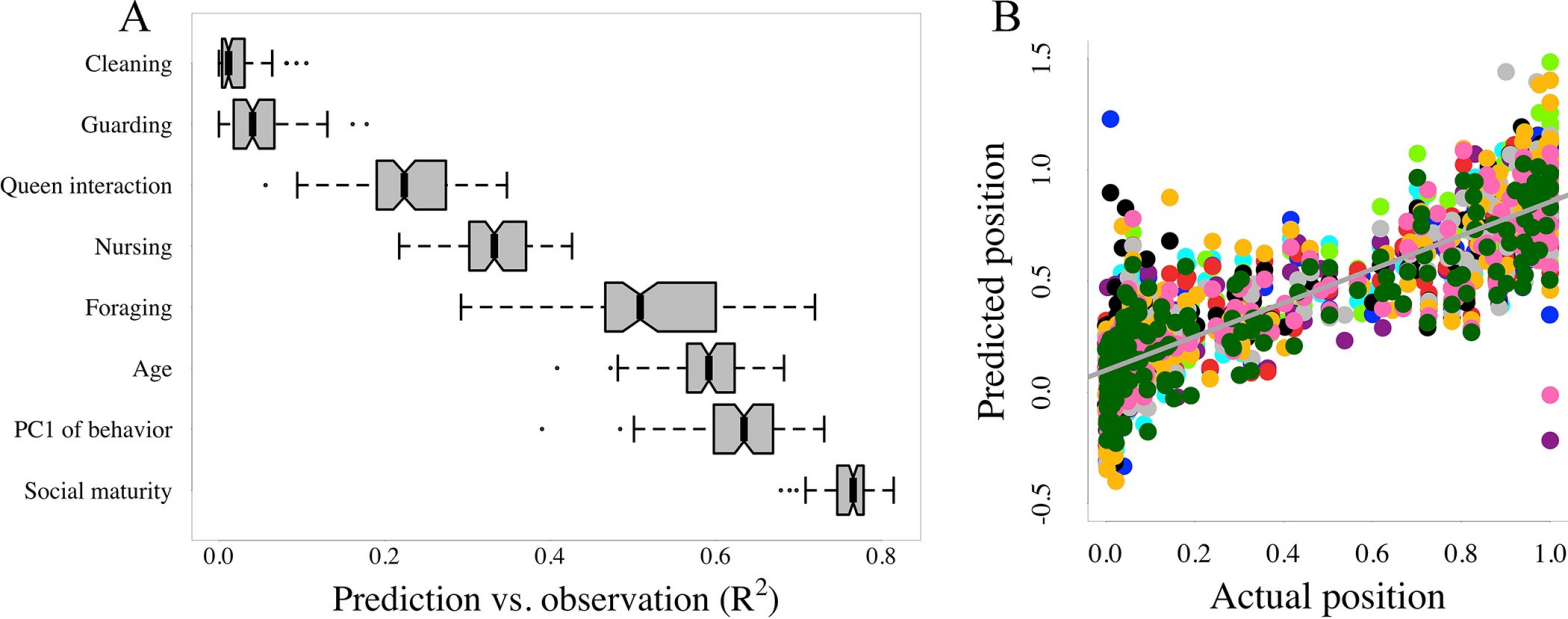
Validation of predictive accuracy. (**A**) Box plots of the R^2^ values between observed and predicted values for the proportion of time spent performing each behavior individually, for position along PC1 of behavioral space, for age, and for social maturity. Black lines indicate median values; boxes and whiskers indicate upper and lower quartiles and 1.5× IQ range, respectively. (**B**) Scatter plot of the predicted versus observed social maturity scores for 10 randomly selected iterations. Color indicates iteration. The code and data used in this figure are available on Zenodo (doi.org/10.5281/zenodo.8043085 - data: “Fig 4A.csv”; code: “05-ML.R”).

To further investigate how brain gene expression patterns relate to social maturity, age, and behavior (both overall behavioral profile and the performance of specific tasks), we used a machine learning approach that is more sensitive to nonlinear associations than the above correlational approaches. We iteratively subsampled half of the worker population at random and trained support vector machine models on their gene expression values and the variable of interest. We then used the model to predict the variable of interest from the brain gene expression data for the other half of the worker population and regressed the predicted values against the observed values to quantify predictive accuracy and, hence, the extent to which the variable of interest is reflected in the brain transcriptome. The highest mean R^2^ between the predicted and observed values (0.76) was obtained for social maturity. The mean R^2^ values between predicted and observed scores were significantly lower for the 7 other aspects of individual biology analyzed (Fig 4; mean R^2^ between the predicted and observed position along PC1 of behavioral space = 0.63; age = 0.59; proportion of time spent foraging = 0.53; nursing = 0.33; tending to the queen = 0.23; guarding = 0.05; cleaning = 0.02; *t* test *p*-values between social maturity R^2^ values and all other R^2^ values all *<*0.01). These results reinforce the suggestion that there is a fundamental link between social network position and brain gene expression and confirm that this link is stronger than that between task behaviors and brain gene expression.

Overall, brain gene expression and microbiota composition correlated more strongly with social network position than with behavior, physical environment, or age. Moreover, while our experiment cannot establish causality or directionality in these relationships, the correlation structure presented here constrains the range of possible causal interactions. If, for example, social interactions were merely a corollary of the spatial distribution of workers (i.e., their physical environments), and if it was physical environment that shaped brain gene expression, then one would expect to see a stronger correlation between the physical environment and brain gene expression than between social environment and brain gene expression. The fact that multiple aspects of behaviorally relevant physiology are more strongly correlated with social interactions than with physical environment, behavior, or age therefore suggests that social interactions may mediate the observed correlations between many aspects of organismal biology and likely play a central role in individual variation in social organisms.

Various factors may limit the generality of these conclusions across species and contexts. First, our experiment was conducted using a eusocial species, and while we would expect the results to hold true for all highly social animals, this remains to be tested. Second, the physical environment was far less complex than those that the ants would naturally experience, which may have reduced the amount of biological variation explained by this variable. Third, composition of the abdominal microbiota of *Camponotus* is atypical in that it is heavily dominated by *Blochmannia*, and in some of our workers constituted exclusively *Blochmannia*. We sequenced to sufficient depth to allow the comparison of the relative abundance of other amplicon sequence variants (ASVs), representing species that were facultatively associated with *C*. *fellah*, and observed correlations between microbiota composition and other biological variables that appeared to be mostly driven by the presence of Acetobacteraceae and other species in foragers but not in nurses (Fig G in S1 Text). However, the dominance of *Blochmannia* and the absence of other bacteria in many individuals may nonetheless reduce the associations between the microbiota composition and the other measured aspects of biology relative to other species.

In conclusion, our study not only highlights the close link between social environments and behavior but also illustrates how social environments relate to behaviorally relevant aspects of physiology, pointing to mechanisms via which individuals can influence each other’s behavior.

## Materials and methods

### Ant colonies

*C*. *fellah* queens were collected following a mating flight in Tel Aviv, Israel, in 2007. The colonies were reared at 27°C under a 12-h/12-h light/dark cycle, provided water and sugar solution ad libitum, and fed weekly with flies and an artificial ant food [34]. For 1 year prior to the experiment (*C*. *fellah* workers generally do not live longer than a year [20]), all newly eclosed workers were paint-marked weekly with a color code indicating their week of birth (Fig 1). *C*. *fellah* queens mate only once, so within colonies, all workers are full sisters [35].

One colony was analyzed in 2018, and 3 colonies were analyzed in 2020. Due to technological advances within the 2 years separating the analyses, the methods used were slightly different. The 2020 methods are reported below, and the differences are detailed in the Supporting information.

### Behavioral tracking

Each colony was created by randomly subsampling approximately 100 workers (approximately 20% of the worker population) from lab stock colonies, along with the queen and approximately 20% of the brood. The colonies were housed in double-chamber setups, with a nest box (170 × 123 mm) kept in constant darkness connected via a plastic tube (internal diameter 19 mm) to a foraging arena (170 × 123 mm) under a 12-h/12-h light/dark cycle. Unique 1.4 mm^2^ matrix barcodes from the ARTag library [36] were fixed to the thorax of each ant using SAUER skin adhesive (Fig 1). These tags weigh a fraction of what ants can carry, and their presence does not alter behavior [37].

Colonies were continuously video recorded for 7 days at 6 frames per second. The tracking system saves video files and the position and orientation of each tag in each frame. Full technical specifications and source code are available at: https://github.com/formicidae-tracker.

### Tracking data processing

Workers that were detected in fewer frames than 2 standard deviations below the colony mean (5 or 6 workers per colony) were excluded from the analysis (“01-MergeData.R”). Each worker’s physical environment was quantified by discretizing the arena into an equilateral grid of hexagonal cells using the mean ant body length as the internal cell diameter and by counting the number of frames in which each worker was detected in each hexagon (“spatial fidelity.ipynb”) [38]. To quantify each worker’s social environment, we inferred pairwise social interactions from the tracking data as in [10]. Head and body regions were annotated for each ant, and interactions were defined as occurring when the head regions of 2 ants overlapped. The total number of pairwise interactions was used to create weighted social networks (Fig 2; “interaction network.ipynb”). This proximity-based definition of social interactions captures trophallaxis (approximately 16% of interactions), grooming (approximately 8% of interactions), and antennation events (approximately 35% of interactions), as well as occasions when 2 individuals move past one another or pause next to one another (approximately 42% of interactions). This high rate of “false positive” interactions has no effect on the data analysis because the frequency of pairwise false positives was well correlated with the frequency of real pairwise interactions, and the absolute numbers are not important because interaction counts are effectively normalized within colonies during analysis. To illustrate this point, we artificially increased the proportion of false positive interactions by comparing the pairwise interaction counts considering only the overlap of head regions (blue in Fig 1B) and the interaction counts when considering also the overlap of body regions (green in Fig 1B), which increases the average number of pairwise interactions from 96 to 260, and which massively increases the false positive rate (e.g., many instances of body-to-body overlap). The R-squared value between the pairwise interaction scores measured in these 2 ways was 0.812.

To quantitatively characterize individual position in the social network (“social maturity”), we used the soft community detection FacetNet (https://c4science.ch/source/facet_unil) [20,39,40]. This approach allows for overlapping social communities by outputting a continuous number in the range 0 to 1 denoting the extent to which a given node belongs to a given community (i.e., where a given worker is positioned between the nurse and forager communities). For this analysis, the number of communities was fixed at two based on previous analyses of *C*. *fellah* social networks [20]. In line with this classification, and consistent with previous results, there was a U-shaped distribution of social maturity scores, with most workers being deeply embedded in one or the other community (Fig D in S1 Text; “02-Main.R”).

### Behavioral annotation

We quantified individual performance of 6 of the most important and commonly performed behaviors. Foraging frequency was automatically quantified as the number of frames in which the individual was detected in the foraging arena divided by the total number of frames in which the individual was detected (“02-Main.R”). The other 5 behaviors were quantified manually, selecting 1 frame every 2 hours (a total of approximately 80 frames) and manually annotating the identities of all workers that were:

1. **Tending the queen:** Positioned near, and orientated toward the queen.
2. **Guarding the nest:** Positioned near, and orientated toward the nest entrance, and stationary.
3. **Nursing the brood:** Stood on the brood pile, or with antennae or mouthparts in contact with brood.
4. **Cleaning:** Stood on the trash pile, or with antennae or mouthparts in contact with the trash pile, or carrying dead ants/ debris.
5. **Engaging in trophallaxis:** Engaged in visible fluid sharing with another individual.

These manually annotated behaviors were not normalized by detection frequency because when an individual’s identity was not detected in the selected frame, we followed the individual through the videos until the identity was resolved. Hence, foraging was bound by 0 and 1, while all other behaviors were bound by 0 and 80, though with different distributions. To give equal weight to all behaviors during dimensionality reduction, each behavior was normalized between 0 and 1 within colonies (“02-Main.R”). PCA of the normalized behavioral data yielded similarly V-shaped plots across the 4 colonies independently, and when data were pooled across colonies (Fig E in S1 Text; “02-Main.R”). For the correlational analyses, including differential expression analysis, PC1 of behavioral space was calculated separately for workers from each colony. For the machine learning–based analysis PC1 of behavioral space was calculated using individuals from all colonies together. The performance of the 6 annotated behaviors mapped onto the social networks in a consistent manner (Fig F in S1 Text; “02-Main.R”).

### RNA extraction, library preparation, and sequencing

Immediately following the tracking experiment, all workers were flash frozen and stored individually in 1.5 ml Eppendorf tubes at −80°C. Brains were later dissected out in 1× PBS and homogenized in 1 ml of TRIzol reagent with ceramic beads in a PRECELLYS Evolution SUPER Homogenizer. Homogenized samples were incubated for 5 min at room temperature (RT) before adding Chloroform (200 μl), vortexing, and incubating for a further 5 min at RT. Samples were centrifuged (25 min at 12,000 rpm and 4°C) and the upper aqueous layer (approximately 500 μl) transferred to a new tube with Isopropanol (650 μl) and Glycogen blue (1 μl, RNAse-free, Invitrogen, 15 mg/ml, #AM9516). Samples were vortexed and incubated overnight at −20°C. Samples were then centrifuged (30 min at full speed at 4°C), the supernatant was discarded, and EtOH (1 ml at 80%) added. Samples were vortexed and centrifuged again (5 min at full speed at 4°C). The supernatant was discarded, and EtOH (1 ml at 70%) added. Samples were vortexed and centrifuged final time (5 min at 12,000 rpm at 4°C). All supernatant was removed, and the pellet was allowed to dry (10 to 15 min) at RT. The pellets were resuspended in nuclease-free water. The KAPA Stranded mRNASeq Library Preparation Kit (#KK8421) was used for library preparation, and samples were sequenced (150 bp, paired-end) using a full S4 FlowCell (4 lanes) on the Novaseq 6000 at the Genomic Technology Facility of the University of Lausanne, yielding 42 *±*7 million (mean *±* SD) reads per sample.

### Gene expression analysis

The transcriptomic reads were mapped to the *C*. *fellah* reference genome (BioProject: PRJNA901066) with STAR v2.7.8a and counted with FeatureCounts using default parameters at all steps (mapping at the gene, not the transcript level) [41,42] (“Mapping.sh”; “Counting.sh”). After mapping and counting, we obtained 38.7 *±* 8.3 million (mean *±* SD) reads per individual.

Before running the differential expression analysis, we filtered out genes with *<*100 reads across all samples (i.e., 1,667 out of 14,664 genes). We used DESeq2 [43] to identify genes that were differentially expressed by each of age, social maturity, and PC1 of the behavioral, microbiota, and physical environment data, as well as for each aforementioned variable when controlling for each other variable, for each of the 4 colonies separately (“03-GeneExpression.R”). In DESeq2, significant differential expression was assessed with a Wald test, and the Benjamini and Hochberg method was used to obtain multiple testing adjusted *p*-values. Genes were considered as differentially expressed when the adjusted *p*-value was *<*0.05.

### Microbiota

Workers were surface sterilized by dipping in 95% ethanol, soaking for 1 minute in 5% bleach, then rinsing with sterilized water. The abdomens were then removed and crushed in PowerBead tubes, and DNA was extracted using the DNeasy PowerSoil kit following the manufacturer’s protocol (https://www.qiagen.com/de/resources/download.aspx?id=91cf8513-a8ec-4f45-921e-8938c3a5490c&lang=en). For each batch of DNA extractions, we also performed blank DNA extractions in which no tissue was added, to control for possible contaminants in the reagents. A mock community composed of 16S rRNA gene plasmids [10] was processed and sequenced along the experimental samples to check for biases introduced during PCR and sequencing. To characterize the microbiota, we amplified the V4 hypervariable region of the 16S rRNA gene following the Illumina 16S metagenomic sequencing preparation guide with minor modifications (https://support.illumina.com/documents/documentation/chemistry_documentation/16s/16s-metagenomic-library-prep-guide-15044223-b.pdf) using primers 515F-Nex (TCGTCGGCAGCGTCAGATGTGTATAAGAGACACCGCGGTAA) and 806R-Nex (GTCTCGTGGGCTCGGAGATGTGTATAAGAGACAGGGACTACHVGGGTWTCTAAT), which contain the adapter sequences for Nextera XT indexes and the primers for the V4 region of the 16S rRNA gene [44]. Briefly, we performed PCR amplifications in a total volume of 25 μl, using 2.5 μl template DNA, 12.5 μl of Invitrogen Platinum SuperFi DNA Polymerase Master Mix, 5 μl MilliQ water, and 2.5 μl of each primer (5 μM). PCR conditions were as follows: 98°C for 30 s followed by 25 cycles of 98°C for 10 s, 55°C for 20 s, and 72°C for 20 s, and by a final extension step at 72°C for 5 min. Amplifications were verified by 2% agarose gel electrophoresis. The PCR products were then purified with Clean NGS purification beads (CleanNA) in a 1:0.8 ratio of PCR product to beads and eluted in 27.5 μl Tris (10 mM, pH 8.5). A second PCR step was performed to append dual-indexes to each sample using the Nextera XT index kit (Illumina). Second-step PCR amplifications were performed in a total volume of 25 μl using 2.5 μl of the products from the first PCR, 12.5 μl of Invitrogen Platinum SuperFi DNA Polymerase Master Mix, 5 μl MilliQ water, and 2.5 μl of each Nextera XT index primer. Thermal cycle conditions were as follows: a first denaturation step at 95°C for 3 min followed by 8 cycles at 95°C for 30 s, 55°C for 30 s, 72°C for 30 s, and a final extension step at 72°C for 5 min. We again purified the PCR products using Clean NGS purification beads in a 1:1.12 ratio of PCR product to beads and eluted them in 27.5 μl Tris (10 mM, pH 8.5). The amplicon concentrations were quantified by PicoGreen, and amplicons were then pooled in equimolar concentration with the exception of the negative control and blank DNA extractions, which were diluted 10×. These controls are intended to show any background noise and contamination, so the same sequencing depth is unnecessary. We verified that the final pool was of the right size using a Fragment Analyzer (Advanced Analytical) and performed sequencing on an Illumina MiSeq sequencer at the Genomic Technology Facility of the University of Lausanne, producing 2 × 250 bp reads.

We obtained a total of 11,552,825 raw sequences, from the 289 abdominal samples, 4 negative PCR controls, 4 mock community samples and 16 blank DNA extractions. Raw sequencing data quality filtered with Trimmomatic [45] using LEADING:3, TRAILING:3, SLIDINGWINDOW:4:15, and MINLEN:180. The quality-filtered data were analyzed with the Divisive Amplicon Denoising Algorithm 2 (DADA2) pipeline (“dada2” package version 1.20.0 in R) [46]. All functions were run using the recommended parameters (https://benjjneb.github.io/dada2/tutorial.html) except that we set randomize = TRUE and nbases = 3e8 at the learnErrors step, and pool = TRUE during the sample inference step. The SILVA database v.138 was used for taxonomy assignments of the identified ASVs. We removed any ASV classified as mitochondria, chloroplast, or Eukaryota (“phyloseq” package v1.36.0 [47], “subset taxa” function). We then used both the “prevalence” and “frequency” methods (method = “either”) in the R package “decontam” v. 1.12.0 [48] to identify and remove contaminants introduced during wet lab procedures, using the negative PCR controls and the blank samples as reference, which allowed us to identify 14 such ASVs (*Renibacterium* sp., *Ralstonia* sp., *Microbacterium* sp., *Leifsonia* sp., *Cutibacterium* sp., *Enhydrobacter* sp., *Gordonia* sp., 2 *Sphingomonas* ASVs, 3 *Methylobacterium-Methylorubrum* ASVs, a Comamonadaceae, and a Finegoldia). The final data set consisted of 10,459,506 reads belonging to 79 ASVs (“Microbiota ABC.csv”; Fig G in S1 Text).

In the analyses, we excluded *Blochmannia*, which is an obligate intracellular endosymbiont of *Camponotus* ants providing essential amino acids and with a likely role in nitrogen recycling [49,50]. This bacterium was present in all individuals (“02-Main.R”).

### Multiplex network visualization and analysis

Euclidean distance was used to calculate pairwise in behavior, brain gene expression, gut microbiota profile (using relative abundances of different ASVs), and physical environment. To generate the layout for each layer in the multiplex network, we used the R package “multinet” [51]. Intralayer edges connect nodes whose similarity/interaction frequency was above the upper quartile, while interlayer edges connect like nodes (i.e., node *i* in layer *a* with node *i* in layer *b*). We used default values for all parameters except for gravity (set to 1) and iterations (set to 1,000). The networks were plotted using python package “mnet.” To compare the correlation between layers, each layer was reduced in complexity to be univariate. Social maturity was used for the social layer and age is de facto univariate. For the other 4 layers, we used PCAs (“02-Main.R”; “04-InterlayerCorr.R”). We extracted PC1 of the microbiota, behavior, and physical environmental data. We extracted PC2 of the gene expression data because all biological variables were best correlated with this principal component, while PC1 was best correlated with extraction batches from the molecular lab (Fig H in S1 Text).

### Prediction with machine learning

To investigate how accurately gene expression data could be used to predict social maturity, behavior, and age, we first used DESeq2 to run a differential expression analysis on the variable of interest, using all samples and controlling for colony identity (model: colony + social maturity; model: colony + PC1 of behavioral data; model: colony + age; model: colony + foraging score, etc.; “05-ML.R”). Genes with fewer than 100 counts across all individuals were removed prior to the differential expression analysis, and genes were considered as differentially expressed when Benjamini–Hochberg adjusted *p*-values were *<*0.05. The support vector machine model (R package “e1071” with type = “eps-regression” and esp = “linear”) was trained using only genes that were differentially expressed by the variable of interest. We used half of all workers for training and the other half for testing and repeated this procedure for 100 iterations. At each iteration, the R^2^ between the predicted and the observed values was calculated and the distribution of these scores over the iterations was used to evaluate prediction accuracy. We used 2-sample *t* tests to compare the distributions of R^2^ values across the hundred iterations between variables.

## Supporting information

S1 Text Fig A. Age distributions. For each of the 4 colonies separately. Fig B. Interlayer correlation networks for each of the 4 colonies separately. Edge width is proportional to edge strength, and layouts are calculated with the Fruchterman–Reingold algorithm. Fig C. Numbers of differentially expressed genes. The numbers of genes differentially expressed by each of the social environment, behavior, age, physical environment, and microbiota alone, and when controlling for each of the other 4 variables for each of the 4 colonies. Genes are considered as significantly differentially expressed when adjusted *p*-values are *<*0.05. Fig D. U-shaped distribution of social maturity scores. In keeping with previous results, there were more workers with extremal than intermediate social maturity scores in all 4 colonies. Fig E. PCA of behavioral data. Left: PCAs for the behavioral data from each of the 4 colonies. Right: PCA of behavioral data from all 4 colonies combined, with workers colored according to their social maturity. Fig F. Behavior mapped consistently onto the social networks. Performance of each behavior was normalized within colony between 0 and 1, and workers are colored according to the task for which they had the highest normalized score. Those categorized as tending the queen (purple) are nearest to the queen (magenta) and surrounded by those categorized as performing brood care (orange). Those categorized as performing cleaning (yellow), trophallaxis (gray)m and guarding (red) are generally located between the 2 social communities, and those foraging (blue) are furthest from the queen. Edge color intensity and width correspond to edge strength. Layouts are calculated with the Fruchterman–Reingold algorithm using R package “iGraph.” Fig G. Overview of the composition of the gut microbiota. (A) colony 1. (B) From left to right: colonies 2, 3, and 4. Samples are ordered by social maturity. Fig H. PCA of gene expression data. The first principal component of gene expression space was well correlated with technical batch effects (extraction groups) and not with any of the biological variables, which all correlated best with the second principal component. R^2^ values are reported.
(PDF)

## Abbreviations

ASV: amplicon sequence variant
LMER: linear mixed effects regression
PCA: principal component analysis
RT: room temperature
