# Supplementary material for: Social network position is a major predictor of ant behavior, microbiota composition, and brain gene expression
Source: PLoS Biol. 2023 Jul 24;21(7):e3002203. doi: 10.1371/journal.pbio.3002203 (PMC10399779; doi:10.1371/journal.pbio.3002203)
Supplement: S1 Text — Fig A. Age distributions. For each of the 4 colonies separately. Fig B. Interlayer correlation networks for each of the 4 colonies separately. Edge width is proportional to edge strength, and layouts are calculated with the Fruchterman–Reingold algorithm. Fig C. Numbers of differentially expressed genes. The numbers of genes differentially expressed by each of the social environment, behavior, age, physical environment, and microbiota alone, and when controlling for each of the other 4 variables for each of the 4 colonies. Genes are considered as significantly differentially expressed when adjusted p-values are <0.05. Fig D. U-shaped distribution of social maturity scores. In keeping with previous results, there were more workers with extremal than intermediate social maturity scores in all 4 colonies. Fig E. PCA of behavioral data. Left: PCAs for the behavioral data from each of the 4 colonies. Right: PCA of behavioral data from all 4 colonies combined, with workers colored according to their social maturity. Fig F. Behavior mapped consistently onto the social networks. Performance of each behavior was normalized within colony between 0 and 1, and workers are colored according to the task for which they had the highest normalized score. Those categorized as tending the queen (purple) are nearest to the queen (magenta) and surrounded by those categorized as performing brood care (orange). Those categorized as performing cleaning (yellow), trophallaxis (gray)m and guarding (red) are generally located between the 2 social communities, and those foraging (blue) are furthest from the queen. Edge color intensity and width correspond to edge strength. Layouts are calculated with the Fruchterman–Reingold algorithm using R package “iGraph.” Fig G. Overview of the composition of the gut microbiota. (A) colony 1. (B) From left to right: colonies 2, 3, and 4. Samples are ordered by social maturity. Fig H. PCA of gene expression data. The first principal component of gene expre [file pbio.3002203.s001.pdf]

Supplementary Information for:

Social network position is a major predictor of ant behaviour, microbiota composition and brain gene expression

Tomas Kay[1,*], Joanito Liberti[1,2], Thomas O. Richardson[1,3], Sean K. McKenzie[1], Chelsea A. Weitekamp[1], Christine La Mendola[1], Matthias Rüegg[1], Lucie Kešnerová[2], Natasha Szombathy[1], Sean McGregor[1], Jonathan Romiguier[1,4], Philipp Engel[2], and Laurent Keller[1,#]

[1]*Department of Ecology and Evolution, University of Lausanne, Switzerland*

[2]*Department of Fundamental Microbiology, University of Lausanne, Switzerland*

[3]*School of Biological Sciences, University of Bristol, United Kingdom*

[4]*Department of Biology and Ecology, University of Montpellier, France*

[#]*Present address: Social Evolution Unit, Cornuit 8, BP 855, Chesières, Switzerland*

[*]*Tomas.kay@unil.ch*

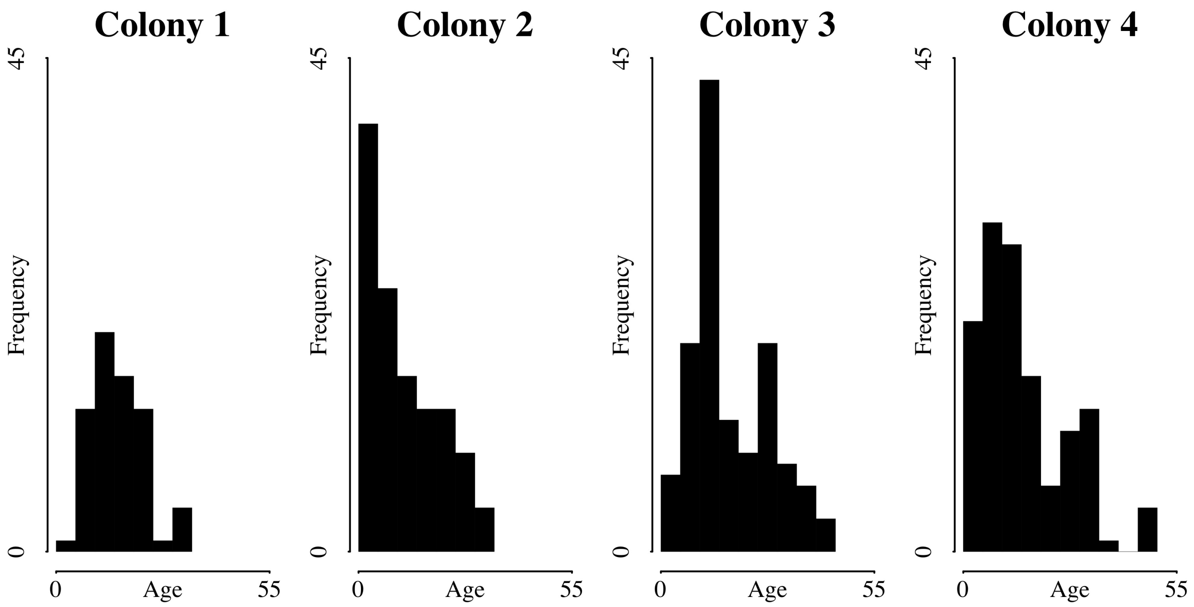

Fig A: **Age distributions** For each of the four colonies separately. The code and data used in this figure are available on Zenodo (data: ‘Fig1C&S1.csv’; code: ‘02-Main.R’)

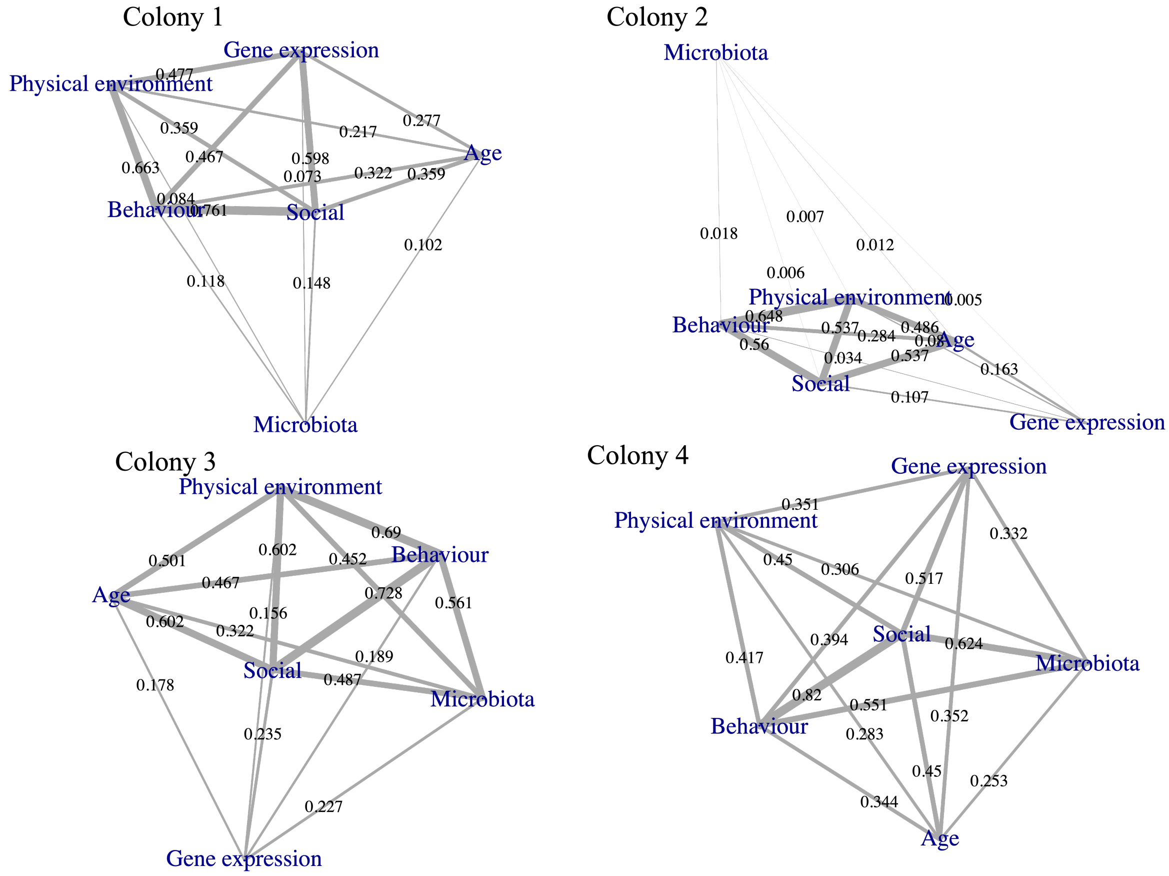

Fig B: **Inter-layer correlation networks** for each of the four colonies separately. Edge width is proportional to edge strength, and layouts are calculated with the Fruchterman-Reingold algorithm[1]. The code and data used in this figure are available on Zenodo (data: all four ‘FigS2...’ csv files; code: ‘04-InterlayerCorr.R’)

	Colony 1	Colony 2	Colony 3	Colony 4
Social	5139	2601	2964	3619
Social controlling for behavior	1194	591	333	433
Social controlling for age	3530	1351	516	1202
Social controlling for physical	3023	69	1020	1201
Social controlling for microbiota	4612	2607	2310	1839
Behavior	4702	2090	2681	3027
Behavior controlling for social	0	24	99	4
Behavior controlling for age	2662	916	561	735
Behavior controlling for physical	1790	12	991	708
Behavior controlling for microbiota	4081	2075	1331	871
Age	3603	1765	2427	3443
Age controlling for social	10	235	131	532
Age controlling for behavior	53	689	333	1097
Age controlling for physical	940	446	483	1555
Age controlling for microbiota	2789	1790	1139	2173
Physical	4068	2534	2708	2719
Physical controlling for social	12	0	61	0
Physical controlling for behavior	16	382	307	26
Physical controlling for age	1857	1365	360	597
Physical controlling for microbiota	3451	2512	2353	692
Microbiota	660	36	1666	2150
Microbiota controlling for social	8	53	5	3
Microbiota controlling for behavior	11	49	4	3
Microbiota controlling for age	3	38	46	412
Microbiota controlling for physical	15	42	294	217

Fig C: **Numbers of differentially expressed genes** The numbers of genes differentially expressed by each of the social environment, behavior, age, physical environment, and microbiota alone, and when controlling for each of the other four variables for each of the four colonies. Genes are considered as significantly differentially expressed when adjusted p-values are < 0.05

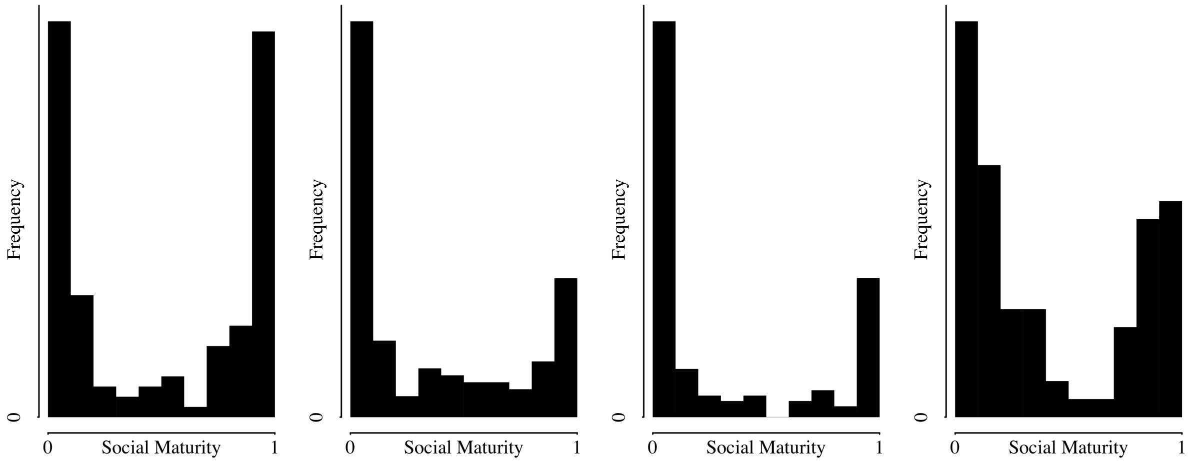

Fig D: **U-shaped distribution of social maturity scores** In keeping with previous results[2] there were more workers with extremal than intermediate social maturity scores in all four colonies. The code and data used in this figure are available on Zenodo (data: 'FigS4.csv'; code: '02-Main.R')

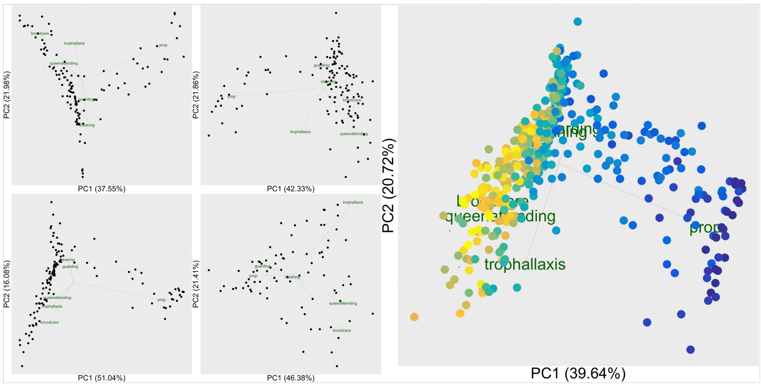

Fig E: **Principal Component Analysis of behavioral data** Left: PCAs for the behavioral data from each of the four colonies. Right: PCA of behavioral data from all four colonies combined, with workers colored according to their social maturity. The code and data used in this figure are available on Zenodo (data: all five 'FigS5...' csv files; code: '02-Main.R')

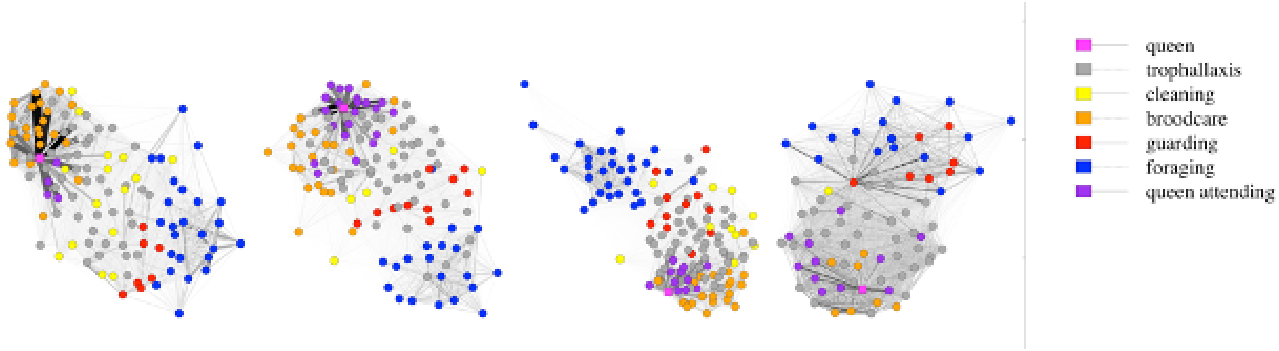

Fig F: **Behavior mapped consistently onto the social networks** Performance of each behavior was normalised within colony between 0 and 1, and workers are colored according to the task for which they had the highest normalised score. Those categorized as tending the queen (purple) are nearest to the queen (magenta), and surrounded by those categorized as performing brood care (orange). Those categorized as performing cleaning (yellow), trophallaxis (gray) and guarding (red) are generally located between the two social communities, and those foraging (blue) are furthest from the queen. Edge color intensity and width corresponds to edge strength. Layouts are calculated with the Fruchterman-Reingold algorithm[1] using R package 'iGraph'[3]. The code and data used in this figure are available on Zenodo (data files: all four 'Fig2A...' csv files; code: '02-Main.R')

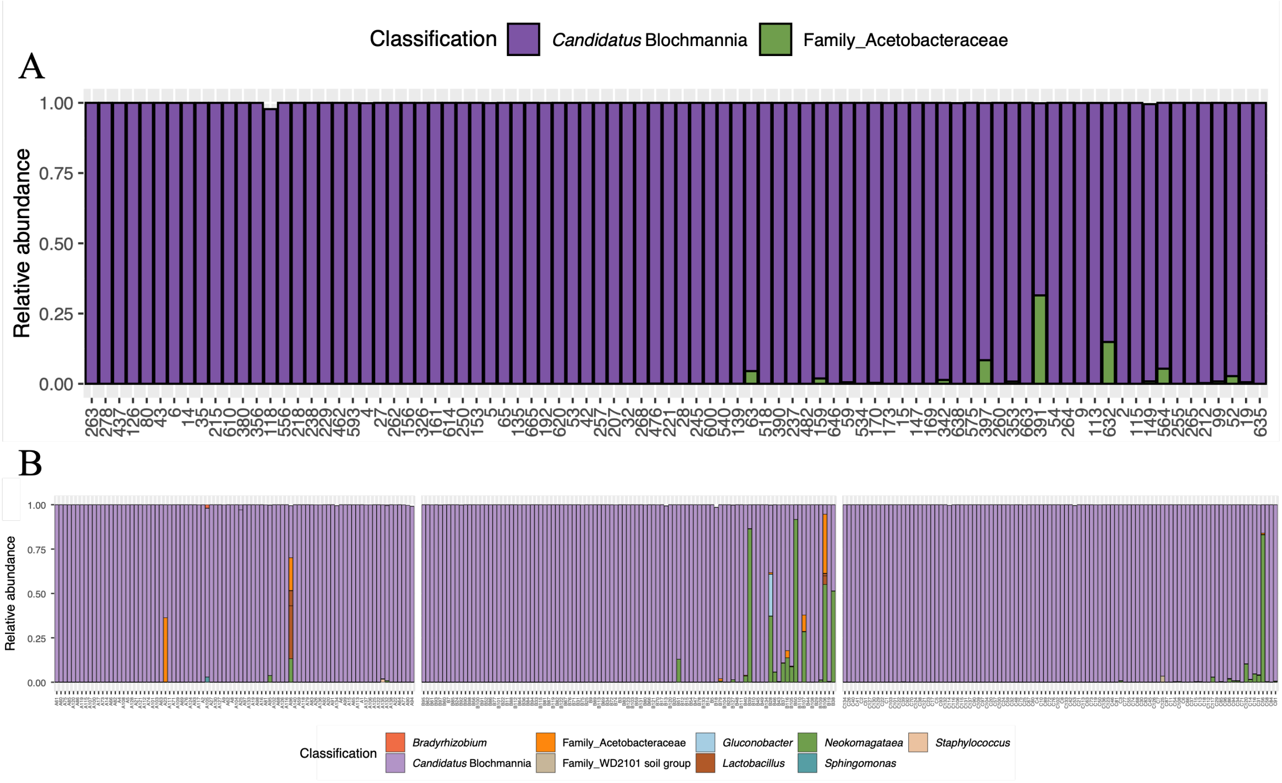

Fig G: **Overview of the composition of the gut microbiota** (A) colony 1; (B) From left to right: colony 2, 3, and 4. Samples are ordered by social maturity. The code and data used in this figure are available on Zenodo (data files: 'FigS7.csv'; code: 'ampliseq_analyses.Rmd' and 'ps_dada2taxa_MultiplexNetwork.rds')

	PC1	PC2
Age	0.017	0.243
Physical	0.013	0.266
Microbiota	0.007	0.16
Social	0.017	0.364
Behavior	0.02	0.271
Extraction	0.312	0.12

Fig H: **Principal Component Analysis of gene expression data** The first principal component of gene expression space was well correlated with technical batch effects (extraction groups) and not with any of the biological variables, which all correlated best with the second principal component. R^2 values are reported

Pilot methodological differences

The main text reports the methods used in 2020 to analyze three of the colonies. The first colony was analyzed in 2018, and the ways in which the original methods differed from the subsequent methods are detailed below.

Behavioral tracking set-up The tracking systems were upgraded between the two experiments. Full technical specifications and source code for the old systems are available at: `https://github.com/laurentkeller/anttrackingUNIL`.

Behavioral tracking data processing In the old post-processing pipeline, physical environments were characterised by discretizing the nest arena into grid-squares and counting the number of visits paid to each square[4]. Social interactions were inferred by modelling each ant as a trapezoid and identifying overlap between the trapezoids of two ants.

Behavioral annotation To quantify individual behavior we systematically sub-sampled the videos, extracting five 10 minute clips of video footage spread evenly between 10:00 and 20:00 for each of the 18 days (total = 15 hours), and annotating every time a worker nursed, cleaned, or trophallaxed (as in **Methods**). Guarding and tending to the queen were inferred automatically:

1. **Guarding the nest:** The number of trajectory fixes where an individual was inside the nest, within $\sim$ 22 mm (two body-lengths) of the nest entrance, aligned towards the nest entrance ($\pm\pi/2$ radians), and stationary (i.e., with a first passage time (FPT) for $\sim$ 22 mm of $>$ 500 seconds. FPT is the time an individual remains within a given radius[5].

2. **Tending the queen:** The number of interactions with the queen.

RNA sequencing Samples were sequenced (single-end) on eight lanes of an Illumina HiSeq 4000.

Gene expression analysis The transcriptomic data were trimmed using trimmomatic v0.36 and reads were then mapped with STAR v2.6.0a[6,7]. After mapping and counting, we obtained 12.8±3.4

million reads per individual (mean±sd).

Microbiota We obtained a total of 3,117,021 raw sequences across 86 samples, two negative PCR controls, two mock community samples and three blank DNA extractions. Quality-filtered data were analyzed with the Divisive Amplicon Denoising Algorithm 2 (DADA2) pipeline ("dada2" package version 1.14.1 in R)[8]. We used the "prevalence" method with a threshold of 0.5 to identify and remove contaminants (removing a total of 7 ASVs: *Enterobacteriaceae sp.*, *Aliicoccus persicus*, *Sphingomonas sp.*, *Staphylococcus sp.*, *Lactobacillus melliventris*, *Acinetobacter sp.*, and *Curvibacter sp.*). The final dataset consisted of 2,564,140 reads belonging to 43 ASVs.

References

[1] Thomas, M. & Edward, M. R. Graph drawing by force-directed placement. *Software: Practice and experience* **21**, 1129–1164 (1991).

[2] Richardson, T. O. *et al.* Ant behavioral maturation is mediated by a stochastic transition between two fundamental states. *Current Biology* **31**, 2253–2260 (2021).

[3] Csardi, M. G. Package 'igraph' (2013).

[4] Crall, J. D. *et al.* Spatial fidelity of workers predicts collective response to disturbance in a social insect. *Nature Communications* **9**, 1201 (2018).

[5] Fauchald, P. & Tveraa, T. Using first-passage time in the analysis of area-restricted search and habitat selection. *Ecology* **84**, 282–288 (2003).

[6] Bolger, A. M., Lohse, M. & Usadel, B. Trimmomatic: a flexible trimmer for Illumina sequence data. *Bioinformatics* **30**, 2114–2120 (2014).

[7] Dobin, A. *et al.* STAR: ultrafast universal RNA-seq aligner. *Bioinformatics* **29**, 15–21 (2013).

[8] Callahan, B. J. *et al.* DADA2: high-resolution sample inference from Illumina amplicon data. *Nature Methods* **13**, 581 (2016).
